# Anti-Toothbreaker: A Novel Low-Budget Device Enabling Contactless Dental Protection and a Forbidden Technique during Direct Laryngoscopy for Endotracheal Intubation

**DOI:** 10.3390/diagnostics13040594

**Published:** 2023-02-06

**Authors:** Sam Razaeian, Helena Kristin Liebich

**Affiliations:** 1 Department of Trauma Surgery, Hannover Medical School, Carl-Neuberg-Straße 1, 30625 Hannover, Germany; 2 Hannover Medical School, Carl-Neuberg-Straße 1, 30625 Hannover, Germany

**Keywords:** orotracheal intubation, dental injury, endotracheal intubation, laryngoscope, trauma surgery

## Abstract

Background: Iatrogenic dental injury is the most common complication of conventional laryngoscopy during orotracheal intubation. The main cause is unintended pressure and leverage forces from the hard metal blade of the laryngoscope. The aim of this pilot study was to introduce and test a novel, reusable low-budget device not only providing contactless dental protection during direct laryngoscopy for endotracheal intubation, but also enabling, in contrast to established tooth protectors, active levering with conventional laryngoscopes for easier visualization of the glottis. Methods: A constructed prototype for intrahospital usage was evaluated by seven participants on a simulation manikin for airway management. Endotracheal intubation was performed with and without the device using a conventional Macintosh laryngoscope (blade size 4) and a 7.5 mm endotracheal tube (Teleflex Medical GmbH, Fellbach, Germany). Necessary time and success of first pass were determined. Degree of visualization of the glottis with and without the device was stated by the participants according to the Cormack and Lehane (CL) classification system and the Percentage of Glottic Opening (POGO) scoring system. In addition, subjective physical effort, feeling of safety regarding successful intubation, and risk for dental injury were queried on a numeric scale between 1 and 10. Results: All participants except one stated that the intubation procedure was easier with usage of the device than without it. On average, this was subjectively perceived as being approximately 42% (range, 15–65%) easier. In addition, time to first pass success, as well as degree of glottis visualization, subjective physical effort, and feeling of safety regarding risk for dental injury, were clearly better with usage of the device. Concerning feeling of safety regarding successful intubation, there was only a minor advantage. No difference in first pass success rate and number of total attempts could be observed. Conclusion: The Anti-Toothbreaker is a novel, reusable low-budget device which might not only provide contactless dental protection during direct laryngoscopy for endotracheal intubation, but also enables, in contrast to established tooth protectors, active levering with conventional laryngoscopes for easier visualization of the glottis. Future human cadaveric studies are needed to investigate whether these advantages also prove themselves there.

## 1. Introduction

Iatrogenic dental injury is the most common complication of conventional laryngoscopy during orotracheal intubation. The prevalence of dental injury is reported in the literature to range from 0.06% to 25%, assuming an underestimation of the actual value [1,2,3,4,5,6,7].

In over 80% of cases, the maxillary incisors are involved. Thus, this iatrogenic injury has not only an esthetic and functional consequence, but also social implications for the patient [1,2,3,4,5,6,7]. It is no coincidence that this injury is the most common reason for medicolegal disputes in the field of anesthesiology in the USA [2,7,8,9].

The main cause is unintended pressure and leverage forces from the hard metal blade of the laryngoscope during orotracheal intubation [2].

Predisposing risk factors include emergency situations, difficult airway (e.g., due to mandibulofacial anomalies or cervical immobilization), and poor dental status (e.g., due to caries, periodontal disease, dentures, crowns, or fixed partial dentures) [2].

Commercially available tooth protectors, such as the EndoraGard™ (Kerr Corporation, Orange, CA, United States), are disposable products made of soft polymer compounds (ethylene–vinyl acetate copolymer), are placed directly on the tooth row, and are not designed to absorb active lever forces. Although levering maneuvers are known to improve visibility and may facilitate the intubation procedure, they are avoided as much as possible during conventional intubation to avoid damage to the upper dentition. Instead, the glottis plane should be visualized by pulling the laryngoscope away from the intubating physician’s body.

The same applies to more rigid thermoplastic protectors, which can be pre-formed with heat prior to use (Intuguard™, SISU, Akervall Technologies Inc.) [8]. These have not only the disadvantage that the necessary heat application of approximately three minutes and the subsequent modeling make its use in an emergency situation unattractive, but the disposable product is also cost-intensive.

The aim of this pilot study was to introduce a novel, reusable low-budget device not only providing contactless dental protection during direct laryngoscopy for endotracheal intubation, but also enabling, in contrast to established tooth protectors, active levering with conventional laryngoscopes for easier visualization of the glottis in the event of difficult airways.

## 2. Materials and Methods

This study was carried out in accordance with the ethical standards of the 1964 Declaration of Helsinki, as updated in 2004. Prior consultation with the local ethical committee of Hannover Medical School took place. An institutional review board statement was not necessary.

### 2.1. Construction of the Anti-Toothbreaker

**Figure 1:**
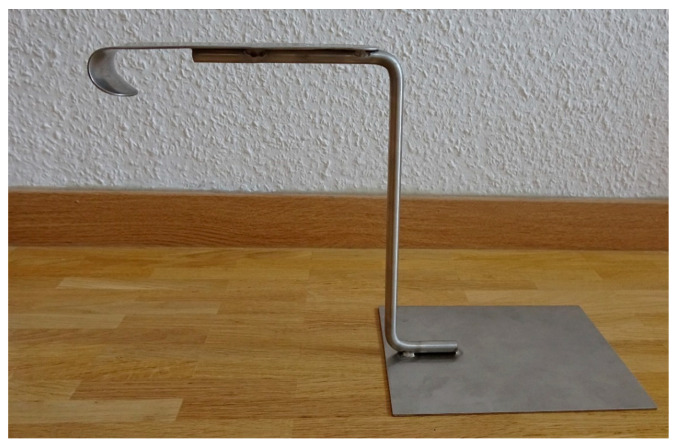
Version A for extrahospital usage in side view.

**Figure 2:**
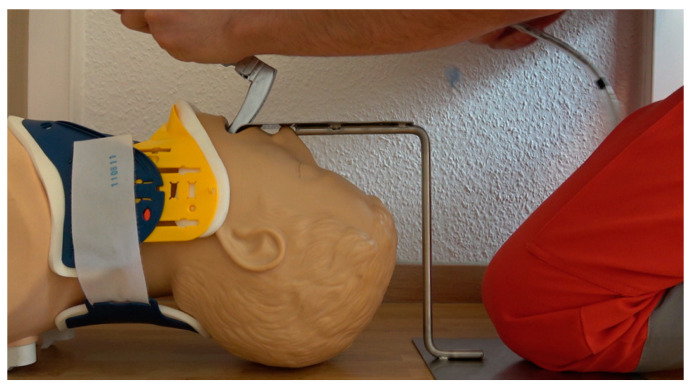
Version A during usage. The round steel is welded and bent with a stainless-steel plate in such a way that this serves as a knee support surface for the emergency physician kneeling at the patient, thus allowing contactless, extraoral transmission of leverage forces.

The device was constructed in the form of a prototype in two different versions. Version A was designed for extrahospital usage with emergency patients lying on the floor and functions autonomously (Figure 1 and Figure 2).

**Figure 3:**
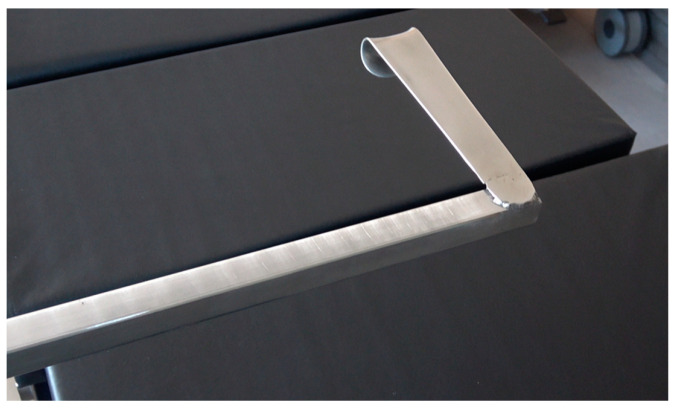
Version B for intrahospital usage.

**Figure 4:**
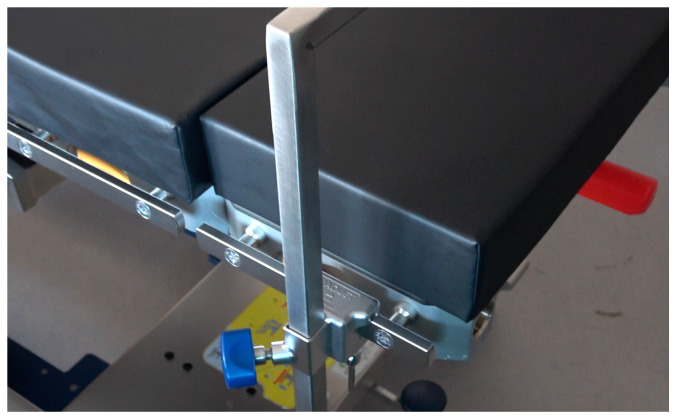
Version B is compatible with commonly used operating tables and holders. Its support and transmission of forces take place outside the oral cavity.

**Figure 5:**
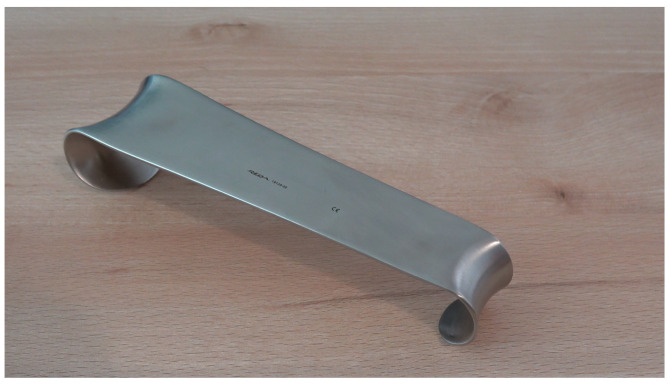
Conventional size 2 surgical Roux retractor (Reda Instrumente GmbH, Tuttlingen, Germany).

Version B was designed for intrahospital usage and is compatible with commonly used operating tables and holders (Figure 3 and Figure 4). Both versions consist of the same mouthpiece, made from a conventional size 2 surgical Roux retractor (Reda Instrumente GmbH, Tuttlingen, Germany) (Figure 5), which is available in different sizes and might anticipate interindividual anatomic differences.

**Figure 6:**
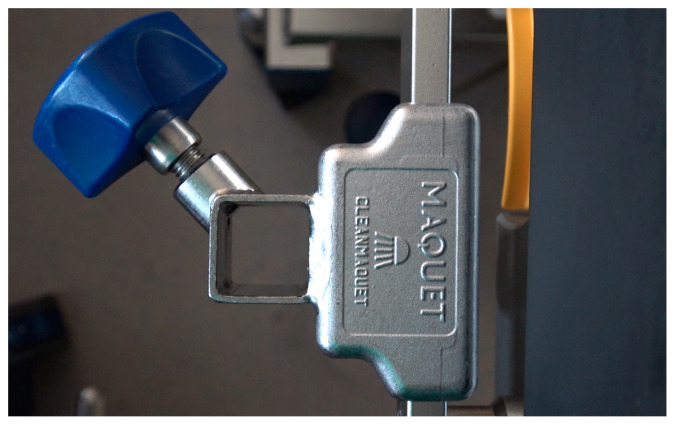
In version B, fixation takes place via a common square tube holder (Maquet GmbH, Rastatt, Germany).

The smaller end of the Roux retractor was bent over and cold-pressed for these purposes. The larger end comes to rest in front of the upper tooth row to be protected. Due to its concave shape, it holds the convex-shaped metal blade of the laryngoscope in a central position and allows a safe lever maneuver without the risk of slipping sideways. In version A, this mouthpiece is welded to V2A round steel (Ø 10 mm). The round steel is welded and bent with a stainless-steel plate in such a way that this serves as a knee support surface for the emergency physician kneeling at the patient, thus allowing sufficient extraoral transmission of leverage forces without further necessary equipment (Figure 2). In version B, the mouthpiece is welded to a rectangular tube in such a way that the construct can be fixed to the operating table in a common square tube holder (Maquet GmbH, Rastatt, Germany) (Figure 6).

While version B is height-adjustable and can be readjusted to individual head sizes, version A is currently not yet height-adjustable. However, this would be technically easy to implement on the round steel by means of a tube connector and is currently under construction.

**Figure 7:**
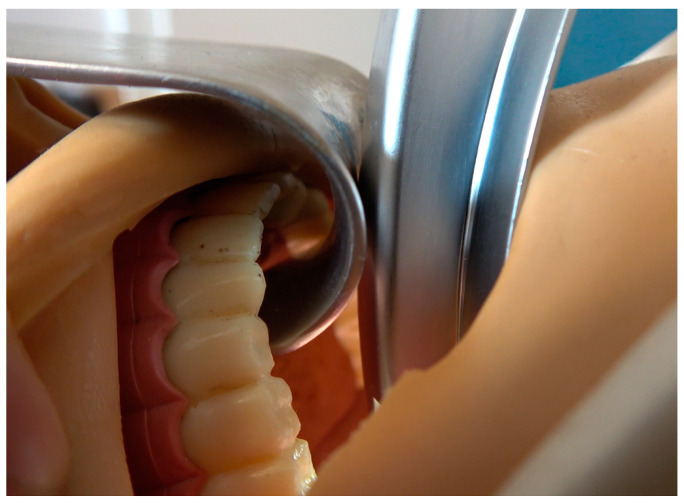
The shape of the mouthpiece allows contactless dental protection and application of active levering forces without the risk of slipping sideways with the blade.

The shape of the mouthpiece and the material properties of the rigid overall construct presented here allow lever forces to be adequately absorbed without having any contact with the upper tooth row (Figure 7).

**Figure 8:**
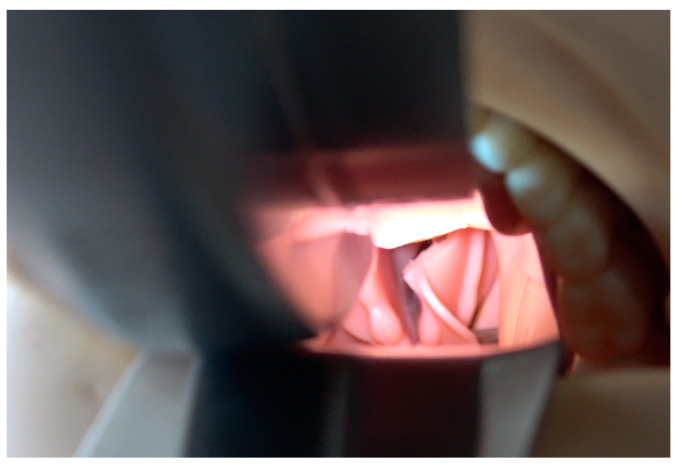
The discreet profile of the mouthpiece also ensures that the device does not unnecessarily obstruct the oral cavity and the intubation path. It should be mentioned that usage of active levering is not mandatory when using this device, as it allows the conventional technique as well.

The support and transmission of forces take place outside the oral cavity, thus avoiding the risk of accidental dislocation of the construct. The discreet profile of the mouthpiece also ensures that the device does not unnecessarily obstruct the oral cavity and the intubation path (Figure 8). At this point, it should be mentioned that usage of active levering is not mandatory when using this device, as it allows the conventional technique as well.

Furthermore, unlike tooth protectors that have been commercially available to date, the device is not only inexpensive to create, but is a reusable product that would be disinfectable and sterilizable due to its material properties.

### 2.2. Testing of the Prototype on a Simulation Manikin

Seven participants with different levels of education were recruited at the authors’ institution to test the device (version B) on a simulation manikin (Laerdal Airway Management Trainer, Laerdal Medical, Stavanger, Norway). In order to complicate direct laryngoscopy, a cervical orthosis (Stifneck Select, Laerdal Medical, Stavanger, Norway) was applied. All participants except one were trauma surgeons regularly involved in airway management during ground- and air-based rescue service as emergency physicians and/or during intensive care activity, with approximately 20 (range, 6–40) self-declared endotracheal intubations per year. The average postgraduate year was 4.8 (range, 3–8). Endotracheal intubation was performed with and without the device using a conventional Macintosh laryngoscope (blade size 4) and a 7.5 mm endotracheal tube (Teleflex Medical GmbH, Fellbach, Germany). Participants had to start with the device first, in order to avoid providing it with any learning curve advantage.

Necessary time and success of first pass were determined by one observer (S.R.). Degree of visualization of the glottis with and without the device was stated by the participants according to Cormack and Lehane (CL) grade and the Percentage of Glottic Opening (POGO) scoring system. In addition, subjective physical effort, feeling of safety regarding successful intubation, and risk for dental injury were queried on a numeric scale between 1 and 10. 10 expressed maximum physical effort, and feeling of safety.

### 2.3. Statistical Analyses

Descriptive statistics, including means and ranges, were calculated. Data analysis was performed with SPSS 26.0 (IBM, Armonk, NY, USA).

## 3. Results

All participants except one stated that the intubation procedure was easier with usage of the device than without it. On average, this was subjectively perceived as being approximately 42% (range, 15–65%) easier. Besides time to first pass success, degree of glottis visualization, subjective physical effort, and feeling of safety regarding risk for dental injury were clearly better with usage of the device. Concerning feeling of safety regarding successful intubation, there was only a minor advantage. In addition, no difference in first pass success rate and number of total attempts could be observed (Table 1).

## 4. Discussion

Over the last decades, many aids have been described for dental protection during direct laryngoscopy for endotracheal intubation. There were different approaches, such as modifying the blade of the laryngoscope [2,5,10,11,12,13,14,15,16,17], padding the blade [5,18,19,20,21] or, probably most popular, using mouthguards [2,5,13,20,22,23,24,25,26,27,28,29,30,31]. All these different approaches differ concerning their technical advantages and disadvantages, as well as economical and sustainability aspects.

Most modified blades have in common that the proximal flange has been reduced [2,5,8,11,14,15,17]. This is a benefit because the risk of potentially damaging the teeth is reduced when the distance between the upper dental arches is increased [2,5,10,13,16,17]. Even though there are a large number of modifications, many of the MacIntosh blades that have been introduced are not in use on a regular basis [2]. It was also shown that intubation with modified laryngoscopes took longer in general, without any significant difference in the potential dental damage [12]. Examples of modified blades are the Callander laryngoscope, the Bizzarri–Giuffrida, the Bucx modification, and the Bellscope blade [2,5,10,11,14,15,20]. For example, one Bizzarr–Giuffrida blade costs approximately EUR 60 [32].

Usage of intraoral plaster, such as Ora-Aid 25 (RUNDAS GmbH, Dinslaken, Germany) and Orahesive, has also been described [5,13,33,34]. This kind of plaster sticks very well on wet surfaces [33] and can be applied to the teeth and gums [5,13,34]. It has minimal impact on visibility [5], and it may reduce the damage to enamel and gums [5,34]. However, it can be difficult to apply these products to the teeth, especially when the ability to open the mouth is limited [13]. In addition, there is also no absolute protection against levering forces [5]. One strip of Ora-Aid 25 costs EUR 3 [35] but is not sustainable, as it is not reusable.

It has also been suggested that a gauze roll or folded tape could be put between the blade and the jaw to protect the teeth [5]. While this is a cheap and easily available option, it might not provide absolute reduction of leverage forces [5], and if it is not properly fixed in its position, it could easily slip out of its place and become an airway hazard. Therefore, it might not be a recommended option for difficult airways. Gauze costs approximately EUR 0.80 per piece [36] and is not reusable.

Using cushions of different materials that are fixed to the blade may also reduce enamel damage [5,18,19,20,21]. It has been suggested to use 3M Microfoam surgical tape for this purpose [18]. This tape is only 1 mm thin, does not interfere with visibility, and allows no sliding of the blade over the teeth [18]. However, this aid might also not provide full protection against levering forces.In addition, if not properly fixed, it could also become an additional airway hazard [5]. Microfoam surgical tape costs approximately EUR 3.8 per meter (2.5 m × 5 m) [37] and is not reusable.

Probably the most reported devices are mouthguards [2,5,13,20,22,23,24,25,26,27,28,29,30,31]. They can be generic, customized, or made out of malleable material. The generic mouthguards have the advantage that they are already commercially available in standard sizes ready to use. However, due to their standard sizes and interindividual anatomic differences the fit is often poor, and they can block the airway [2,5,13,20,23,29]. In addition, evidence suggests that they also might not provide reliable protection [5,23,29].

The customized ones are manufactured by dentists and dental laboratories. They provide significant protection against dental trauma through absorbing and spreading forces [5,22,23,25,38]. However, because the production process can take quite long, it is not useful in emergency situations [13,23,38]. Malleable dental guards are mostly made out of materials that soften up when placed in hot water and can then be molded to the patients’ teeth [24,27,28]. This can also be performed at the bedside [24,27,28], which is quite an advantage. However, they still need a couple of minutes to mold, so they also might not be useful in emergency situations [13,23]. Costs range from EUR 6 to EUR 30 for the generic ones [39]. Prices for the malleable ones depend on the product [24,40]. For example, Intuguard™ costs about EUR 30 per piece [40]. For customized mouthguards, there no published data could be found.

Depending on the product, it may be reusable for the same patient [25,31]. This applies in particular tocustomized mouthguards. Because the forces are mostly not absorbed entirely, but transferred to the adjoining teeth [5], it can still cause harm to the maxillary teeth. Only one study has shown that the Intuguard™ device apparently might transfer zero force to the teeth [27]. Most of the currently existing devices have in common that they are expensive, are not sustainable, because not reusable, pose a risk to dislocate and hinder the intubation pathway due to intraoral attachment, or are not designed to provide full protection against active leverage forces.

In contrast to that, the novel low-budget device presented here might anticipate these features. Firstly, it is cheap to produce [41], and the unique shape of the mouthpiece is already commercially available in different sizes. Secondly, due to its material properties, it is reusable, as it is disinfectable and sterilizable. Thirdly, there is no risk of accidental dislocation due to its extraoral attachment. In addition, at the same time, this feature enables transmission of forces without having any contact with the upper tooth row, allowing even active levering for easier visualization of the glottis during direct laryngoscopy. To the best of our knowledge with extensive review of the literature, a device utilizing these principles does not exist yet.

Nevertheless, there are several limitations to consider. The findings of this investigation are based in the context of a pilot study on a training simulator. It remains unclear whether the observed advantages would also apply to an in vivo setting. In addition, the introduced device was only evaluated by seven physicians in different postgraduate training years. As the sample size is too small, a meaningful subgroup analysis concerning their different levels of experience cannot be performed. Even though the majority of participants were regularly involved in airway management during ground- and air-based rescue service and/or intensive care activity, none of them was a trained anesthesiologist, which must be considered as a major limitation of this study. It remains questionable whether usage of the device would have a benefit even if experienced anesthesiologists had participated. Lastly, it remains unclear to what extent local pressure can damage the laryngopharynx by enabling active levering. At least we believe that safety could benefit if the intraoral side of the Roux retractor, which comes close to the hard palate, is covered with thin silicone rubber.

## 5. Conclusions

The Anti-Toothbreaker is a novel, reusable low-budget device which might not only provide contactless dental protection during direct laryngoscopy for endotracheal intubation, but also enables, in contrast to established tooth protectors, active levering with conventional laryngoscopes for easier visualization of the glottis. Future human cadaveric studies are needed to investigate whether these advantages also prove themselves there.

## Figures and Tables

**Table 1:** Results of prototype testing. Conv. = Conventional direct laryngoscopy. CL = Cormack and Lehane (CL) classification system.

Participant No.	First Pass Success		Number of Attempts		Time to First Pass Success in Seconds		CL		POGO-Score		Subjective Physical Effort		Subjective Sense of Safety Regarding Successful Intubation		Subjective Sense of Safety Regarding Iatrogenic Dental Injury	
	conv.	with device	conv.	with device	conv.	with device	conv.	with device	conv.	with device	conv.	with device	conv.	with device	conv.	with device
1	1	1	1	1	69	40	1	2	75	25	8	6	8	7	7	10
2	2	1	2	1	98	15	2b	1	10	90	7	1	5	10	6	10
3	1	2	1	2	16	24	2	1	30	80	4	6	9	7	6	9
4	1	1	1	1	20	21	2b	1	60	90	7	3	6	8	3	6
5	1	1	1	1	22	31	2a	2a	50	50	4	4	9	8	2	10
6	1	1	1	1	21	18	3	2	70	90	8	5	5	6	5	10
7	1	1	1	1	18	18	2	1	70	100	8	5	10	10	7	10
Mean					37.7	23.9			52.1	75.0	6.6	4.3	7.4	8	5.1	9.3

## Data Availability

Not applicable.
